# The Effects of Die Temperature and Screw Speed on Extruded Pulse Flours and Their Application in Bread Production

**DOI:** 10.1002/fsn3.70801

**Published:** 2025-08-25

**Authors:** Kübra Tuluk, Burak Altınel, Neslihan Bozdogan, Sebnem Tavman, Seher Kumcuoglu

**Affiliations:** ^1^ Department of Food Engineering, Graduate School of Natural and Applied Sciences Ege University İzmir Türkiye; ^2^ Department of Food Engineering, Faculty of Engineering Ege University İzmir Türkiye

**Keywords:** bread, broad bean flour, extrusion, mung bean flour

## Abstract

Extrusion is an innovative technology for improving the techno‐functional and nutritional properties of pulse flours. This study aimed to optimize extrusion conditions for broad bean and mung bean flours and to assess their potential in bread making. Die temperature (135°C–165°C) and screw speed (200–300 rpm) were optimized using response surface methodology, with water absorption index (WAI), phytic acid (PA), and insoluble dietary fiber (ISDF) as response variables. Optimal conditions were found to be a 165°C die temperature and a 200 rpm screw speed for both pulses. Die temperature and screw speed had a significant effect on WAI, PA, and ISDF values. Flours obtained under optimum conditions showed the following changes: in mung bean, WAI increased by 50%, whereas PA and ISDF decreased by 59.5% and 30.9%, respectively; in faba bean, WAI increased by 33.69%, whereas PA and ISDF decreased by 45.27% and 29.68%, respectively. Extrusion disrupted starch crystallinity and changed protein‐carbohydrate structures as observed by XRD, FTIR, and DSC analyses. Incorporation of pulse flours affected the rheological properties of the bread dough, causing a decrease in viscous and elastic responses. In bread making trials, wheat flour was substituted with pulse flours at 12.5% and 25%. Both substitution levels reduced bread volume and increased crumb hardness. In conclusion, it is shown that extrusion is an effective method for modifying the functional properties of pulse flours, and the use of optimized extrusion as a tool to develop novel functional pulse‐based ingredients.

## Graphical abstract

Extruded Pulse Flours: Breadmaking Applications.

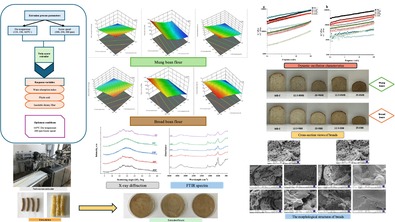

## 1 Introduction

Extrusion is a thermal‐mechanical processing technique widely used in food manufacturing, such as the production of breakfast cereals, snacks, pasta, and plant‐based ingredients, due to its energy efficiency, high production capacity, versatility, and ability to improve nutritional and functional properties (Kesselly et al. 2023). Key extrusion parameters such as temperature, screw speed, and moisture influence product quality and functionality (Pasqualone et al. 2021). Extrusion induces structural and biochemical transformations—such as protein denaturation, starch gelatinisation, and increased soluble fiber—enhancing techno‐functional properties and reducing antinutritional compounds like phytic acid, tannins, and trypsin inhibitors (Pasqualone et al. 2021; Faliarizao et al. 2024).

As dried edible seeds, pulses serve as rich sources of plant‐based protein, dietary fiber, complex carbohydrates, essential minerals, and various bioactive phytochemicals. Due to their favorable nutrient profile and functional properties, pulses play a key role in promoting sustainable and health‐supportive diets. Compared to cereal grains, pulses generally contain higher protein levels and a more favorable amino acid composition (Faliarizao et al. 2024).

Among pulses, mung bean is notable for its high digestible protein content, antioxidant capacity, and relatively short cooking time, making it suitable for various food applications (Tang and Sun 2014). Faba bean, on the other hand, has attracted increasing interest due to its high protein concentration, starch functionality, and potential use in the development of high‐protein and gluten‐free food products (Multari et al. 2015). These properties make both pulses promising candidates for innovative food processing approaches aimed at improving nutritional quality and functionality.

Despite their nutritional advantages, pulses also contain certain antinutritional factors—such as phytic acid, lectins, tannins, and protease inhibitors—that can impair protein digestibility and reduce nutrient bioavailability when consumed in raw or inadequately processed forms (Gupta et al. 2015). To mitigate these limitations and enhance the functional and nutritional quality of pulses, a wide range of processing techniques—spanning physical, chemical, and biological approaches—have been studied (Dhull et al. 2022). Among these, extrusion has recently been identified as a particularly promising method for improving the nutritional profile and techno‐functional properties of faba beans (Salvador‐Reyes et al. 2024) and mung beans (Mathad et al. 2025).

Extrusion process imparts desirable techno‐functional properties to the resulting flours, including improved water absorption and emulsification capacity, which enhance their applicability in diverse food systems. Due to its rapid, continuous, and cost‐efficient nature, extrusion is widely regarded as suitable for industrial‐scale pulse processing (Salvador‐Reyes et al. 2024). Several studies have reported that extrusion enhances the functional properties of pulse‐based flours; increased water holding capacity and water solubility were observed in extruded pea (Luo and Koksel 2020) and kidney bean flours (Alonso et al. 2000). It was also observed the effects of extrusion cooking on yellow pea flour to modify its techno‐functional properties, resulting in protein denaturation and starch gelatinization, which in turn increased water solubility, water binding capacity, and cold viscosity, whereas decreasing emulsion capacity, emulsion stability, and final viscosities (Fernandes et al. 2025).

The primary objective of the present study was to optimize the extrusion process parameters—specifically screw speed and die temperature—and to evaluate their effects on the functional, structural, and pasting properties of mung bean and broad bean flours. Additionally, the study aimed to investigate the incorporation of optimally extruded flours into bread formulations at varying substitution levels.

## 2 Materials and Methods

### 2.1 Materials

Raw mung bean ( *Vigna radiata* L.) flour (RMF) and raw broad bean ( *Vicia faba* L.) flour (RBF) were purchased from the local market. RMF and RBF were sieved using a 500 μm sieve to get flour with reduced particle size, and the flour passed under the sieve was used. Wheat flour (WF) was supported by Söke Değirmencilik San. ve Tic. A.Ş., Aydın, Türkiye. The other ingredients incorporated in bread making trials, including salt and instant yeast, were sourced from the local market. All chemicals and reagents utilized in this study met analytical‐grade standards.

### 2.2 Proximate Properties of Flours

The moisture content was measured by applying ICC Standard Method No. 110/1 (2003), utilizing a laboratory‐type oven (Köttermann Typ 2702, Germany). Ash content was measured in accordance with ICC Standard Methods No. 104/1 (2003), utilizing an ash oven (Carbolite, Type: ELF 11/68, England). Protein content was determined in accordance with ICC Standard Methods No. 105/2 (2003), utilizing an automatic distillation system (Gerhardt Vapodest 20, Type VAP 20, Germany). Total dietary fiber content was assessed following AOAC Method 991.43. Color values (L*, a*, b*) were measured using a color measurement device. Flour blends' water absorption capacity was measured in accordance with ICC Standard Methods No. 115/1 (2003), utilizing a farinograph fitted with a 300 g stainless steel mixing bowl (Mod. PL Nr. 952065, Brabender, Duisburg, Germany).

### 2.3 Experimental Design

The central composite design (CCD) was applied to design the experimental setup, incorporating two independent variables: die temperature (135°C–165°C) and screw speed (200–300 rpm). The conditions for each variable were set in accordance with the results of preliminary trials. Response Surface Methodology (RSM) was employed to design experimental parameters aimed at optimizing extrusion conditions to produce extruded mung bean flour (EMF) and extruded broad bean flour (EBF). To assess the effect of independent variables on the water absorption index (WAI), phytic acid (PA), and insoluble dietary fiber (ISDF) of extruded flours, 13 experimental runs were performed based on the face‐centered CCD matrix generated using Design‐Expert (Version 12; Stat‐Ease Inc., MN, USA), consisting of five center points. Quadratic equations were used to model the independent variables as functions of the dependent variables. The adequacy of the model fit was evaluated using the lack of fit. Finally, the calculated response values were experimentally confirmed.

### 2.4 Extrusion Process

Extrusion was performed using a laboratory‐type corotating twin‐screw extruder (Feza Machine Co. Ltd., Istanbul, Türkiye) equipped with a 25 mm barrel diameter, a L/D ratio of 25:1, and a single circular die of 3 mm. The barrel of the extruder was divided into four separate zones. The temperatures of the first three zones were kept constant at 60°C, 100°C, and 130°C (from inlet to die) throughout all runs. The temperature of the fourth zone and die (135°C–165°C) and screw speed (200–300 rpm) were changed following the experimental design. The flour's moisture content was kept constant at 18%. The flours were conveyed into the extrusion barrel at a feed rate of 10 kg/h. The extrudates were allowed to cool to room temperature and subsequently dried at ambient temperature overnight. The dried extrudates were processed in a hammer mill (Falling Number AB Type 121, Stockholm, Sweden) to obtain extruded flour, which was then stored in polyethylene bags at room temperature for further analysis. The following analyses used non‐extruded (raw, as the control) and extruded flours passing through the 250 μm sieve.

### 2.5 Determination of Product Responses

The following analyses were performed using non‐extruded (raw, as the control) and extruded flours that passed through a 250 μm sieve.

#### 2.5.1 Water Absorption Index (WAI)

The WAI was measured and calculated according to the method described by Anderson et al. (1969). 2.5 g of flour sample was dispersed in 30 mL of distilled water in a water bath at 30°C and stirred for 30 min. The dispersion was centrifuged at 3000 *g* for 10 min. Afterwards, the supernatant was decanted into a pre‐weighed moisture dish to determine the solid content. The supernatant was evaporated overnight at 105°C. WAI was calculated utilizing the following equation:

(1) $$\mathrm{WAI}\;\left(\frac{g}{g}\right)=\frac{\text{Weight of sediment}}{\text{Sample weight}}$$

#### 2.5.2 Phytic Acid (PA)

The PA content was determined as described in Haug and Lantzsch (1983). 0.06 g of flour was stirred in 10 mL of 0.2 N HCl solution for 1 h at room temperature for PA extraction. The extract (0.5 mL) was mixed with 1 mL of ferric solution in the boiling water bath for 30 min. Then, the solution was cooled in an ice bath for 15 min, followed by centrifugation at 3000 *g* for 30 min. 1 mL of the supernatant was mixed with 2 mL of the 2,2′‐bipyridine solution, and the absorbance was recorded at 519 nm using a spectrophotometer.

#### 2.5.3 Insoluble Dietary Fiber (ISDF)

The content of insoluble dietary fiber was quantified using a dietary fiber assay kit (Megazyme) following the AOAC Method 991.43 (AOAC 1992).

### 2.6 Optimization and Model Validation

The experiments were designed and analyzed using the Design‐Expert software (Version 13; Stat‐Ease Inc., MN, USA) to evaluate the impact of independent variables on the responses. The model *F*‐value and lack‐of‐fit *F*‐value were examined to determine the model's significance. RSM analysis was employed to create a second‐degree model for the responses, with the coefficient of determination (*R* ^2^), adjusted *R* ^2^, predicted *R* ^2^, and lack‐of‐fit tests used to assess the model's accuracy. RSM analysis focused on WAI, PA, and ISDF as the selected responses. All independent variables were set within specified ranges and given equal importance to optimize extrusion. Higher WAI values were preferred, whereas lower values for PA and ISDF were considered optimal. The final steps of RSM involved optimization and verification. The Design Expert Software point prediction was utilized to determine the best extrusion parameters. The parameters with the highest desirability value were chosen as the optimal settings. The effectiveness of the models was confirmed by applying the selected optimum parameters using the Design Expert software (Version 12; Stat‐Ease Inc., MN, USA). The response variables WAI, PA, and ISDF were characterized after developing and analyzing the experimental batch of the optimum parameters using the independent factors.

### 2.7 Physicochemical Properties of Extruded Flours

All analyses given below were performed using flours obtained at optimum extrusion parameters.

#### 2.7.1 X‐Ray Diffraction (XRD)

The samples' crystallinity was assessed using an X‐ray diffractometer (MiniFlex 600; Rigaku Americas Corp., The Woodlands, TX, USA), which was equipped with a Cu Kα radiation source. The scattering angle (2θ) was set between 4° and 40° with a 0.05° step length.

#### 2.7.2 Fourier Transform Infrared Spectroscopy (FTIR)

The infrared spectra of flours used as raw materials in bread production were recorded using an infrared spectrometer (Fourier Transform Infrared Thermo Scientific NICOLET iS10) between the wave number range of 400–4000 cm^−1^.

#### 2.7.3 Thermal Properties

Thermal characteristics, namely initial temperature (*T*_o_) and peak temperature (*T*_p_) of flour samples, were analyzed using differential scanning calorimetry (Q2000; TA Instruments, USA). Briefly, a 12 mg sample was blended with distilled water (30:70) and put into hermetically sealed pans at room temperature. The samples were subsequently loaded into a standard DSC cell along with a reference blank cell after 2 h, and they were assessed from 25°C to 120°C at a rate of 5°C/min (Kotsiou et al. 2021).

### 2.8 Pasting Properties

The pasting properties of flours and their blends used in bread production were assessed using a modified method based on Jeong et al. (2017). The analyses were conducted with a rheometer (DHR3; TA Instruments, USA) equipped with a starch pasting cell. In brief, 2.5 g of the sample was mixed with 22.5 mL of distilled water, placed in the pasting cell, and maintained at 50°C for 1 min. Subsequently, the slurry was heated to 90°C at a rate of 3°C per minute and held at this temperature for 2.5 min. Afterwards, the sample was cooled to 30°C at the same rate and kept at 30°C for 2 min. The pasting profile parameters measured included pasting temperature, initial peak viscosity (IPV), trough viscosity (hot gel viscosity, HPV), and final viscosity (cold gel viscosity, CPV). The breakdown viscosity (BV, IPV minus HPV) and setback viscosity (SV, CPV minus HPV) were determined.

### 2.9 Rheological Characterization of Bread Dough

The rheological characterization of dough samples was performed using a stress‐controlled hybrid rheometer (DHR3, TA Instruments, USA) at a controlled temperature of 25°C following the attainment of an axial normal force of 1 N. To mitigate slippage during measurements, a parallel plate geometry featuring a sandblasted surface with a diameter of 40 mm was employed, maintaining a plate gap of 2 mm. To prevent drying of the samples during the analysis, silicone oil was applied to shield the samples from air exposure, following the removal of excess material before analysis. The linear viscoelastic region was delineated through strain sweep analyses conducted at a frequency of 1 Hz, with strain values ranging from 0.001% to 1%. Subsequently, frequency sweep analyses were made over a frequency range of 0.01–100 Hz at a constant strain amplitude of 0.02%.

### 2.10 Bread Making Method

The bread making method in Coda et al. (2017) was based on slight modifications in our bread making trials. The following ingredients were used in bread making trials: WF, RMF, EMF, RBF, EBF at 12.5% and 25% substitution levels; water (in accordance with the water absorption capacity measured via farinograph), 1.2% salt (w/w, flour weight basis), 1.05% instant yeast (w/w, flour weight basis).

In bread making trials, a laboratory‐type mixer (ISM 10, İnoksan, Türkiye) was used. In the mixer, the dough was kneaded for 2 min at a low speed (75 rpm) and for 8 min at a high speed (150 rpm). We determined the optimal kneading conditions (mixer speed and mixing time) by pre‐studies. After kneading, the dough was leavened for 20 min at 32°C–34°C and 75%–80% relative humidity using a proofing cabinet (FGM 100, İnoksan, Türkiye). Following the leavening process, the dough was portioned into four equal portions (175 ± 0.50 g each), hand molded, transferred into pans, and proofed for 60 min at 32°C–34°C and 75%–80% relative humidity in a proofing cabinet. After proving, baking was performed using a ventilated oven (FBE 010, İnoksan, Türkiye) for 15 min at 200°C. The optimal baking time and temperature were determined through preliminary experiments. After baking, the breads were allowed to cool at room temperature for 1 h before bread quality analyses. Bread making trials were conducted in triplicate.

### 2.11 Bread Quality Characteristics

#### 2.11.1 Bread Quality Analysis

The loaf volume of bread was determined with the VolScan Profiler (VSP600; Stable Micro Systems, Surrey, England). The specific volume refers to the loaf volume divided by its mass (cm^3^/g). The percentage of baking loss was calculated based on the difference between the dough weight before baking and the bread weight after baking. AACC Method 44‐15A (2000) was used to determine the moisture content of bread. The hardness of the bread crumb was evaluated through the use of a texture analyzer (TA.XT Plus; Stable Micro Systems, Surrey, England) with a 20‐mm‐diameter cylindrical probe (P/20R). Following slicing, two central slices from each bread were utilized for texture analysis. The following parameters were used in the texture analysis: 40% compression rate, 5.0 g trigger force, and 5 s for the interval time between two compressions. The pretest, test, and post‐test speeds were set at 1.00, 1.70, and 10.00 mm/s, respectively.

#### 2.11.2 Scanning Electron Microscopy (SEM)

The microstructure of the produced breads was determined using SEM (Zeiss EVO‐HD‐15, Jena, Germany). The analysis was conducted at Dokuz Eylul University Science and Technology Application and Research Center, Central Research Laboratory. The samples were prepared by cutting them 1 cm × 1 cm horizontally and longitudinally and subjecting them to freeze‐drying.

### 2.12 Statistical Analysis

The data were assessed using an analysis of variance (ANOVA). Differences between individual means were evaluated using the Duncan Comparison Test (*p* < 0.05) (SPSS Inc., Version 15.0, USA).

## 3 Results and Discussion

### 3.1 Effect of Extrusion Conditions on Extruded Flour Properties

The results obtained for the responses (WAI, PA, ISDF) are presented in Table S1. The effect of extrusion conditions on extruded flour properties with coded and actual experimental levels from the central composite design, as well as the experimental results for WAI, PA, and ISDF of the extruded flours compared to the raw flours, are presented in Table 1.

**TABLE 1:** Effect of extrusion conditions on extruded flour properties.

Run	Independent variables		Product responses					
	DT (°C)	SS (rpm)	Mung bean			Broad bean		
			WAI (g/g)	PA (mg/100 g)	ISDF (%)	WAI (g/g)	PA (mg/100 g)	ISDF (%)
	X_1_	X_2_						
Raw			2.30 ± 0.04 ^g^	1673.06 ± 45.14 ^a^	17.70 ± 0.36 ^a^	2.82 ± 0.07 ^i^	2572.60 ± 58.65 ^a^	22.84 ± 0.46 ^a^
1	135 (−1)	200 (−1)	3.70 ± 0.05 ^a^	1244.53 ± 45.23 ^d^	16.34 ± 0.29 ^b^	4.05 ± 0.03 ^a^	2010.57 ± 55.99 ^d^	18.75 ± 0.38 ^b^
2	135 (−1)	250 (0)	3.56 ± 0.01 ^b^	1294.24 ± 45.45 ^c^	15.34 ± 0.28 ^c^	3.73 ± 0.02 ^c^	2076.34 ± 63.48 ^c^	18.52 ± 0.40 ^b^
3	135 (−1)	300 (+1)	3.32 ± 0.01 ^d^	1386.21 ± 49.51 ^b^	14.16 ± 0.31 ^d^	3.44 ± 0.01 ^f^	2197.42 ± 58.85 ^b^	18.30 ± 0.41 ^bc^
4	150 (0)	200 (−1)	3.57 ± 0.04 ^b^	870.78 ± 24.01 ^g^	13.80 ± 0.27 ^de^	3.93 ± 0.07 ^b^	1754.44 ± 57.52 ^g^	17.94 ± 0.27 ^bcd^
5	150 (0)	250 (0)	3.38 ± 0.03 ^cd^	917.81 ± 23.64 ^f^	13.10 ± 0.41 ^fg^	3.59 ± 0.02 ^e^	1815.38 ± 56.59 ^f^	17.56 ± 0.34 ^cd^
6	150 (0)	250 (0)	3.41 ± 0.05 ^cd^	924.34 ± 22.30 ^f^	13.22 ± 0.27 ^ef^	3.66 ± 0.02 ^d^	1810.77 ± 58.64 ^f^	17.38 ± 0.48 ^cde^
7	150 (0)	250 (0)	3.37 ± 0.04 ^cd^	926.50 ± 31.18 ^f^	13.05 ± 0.28 ^fg^	3.64 ± 0.01 ^de^	1801.27 ± 51.03 ^f^	17.45 ± 0.32 ^cde^
8	150 (0)	250 (0)	3.43 ± 0.02 ^c^	925.37 ± 26.82 ^f^	13.01 ± 0.23 ^fg^	3.61 ± 0.01 ^de^	1802.77 ± 44.27 ^f^	17.48 ± 0.43 ^cde^
9	150 (0)	250 (0)	3.40 ± 0.04 ^cd^	926.08 ± 32.07 ^f^	13.14 ± 0.25 ^efg^	3.63 ± 0.02 ^de^	1804.36 ± 46.32 ^f^	17.27 ± 0.38 ^de^
10	150 (0)	300 (+1)	3.18 ± 0.05 ^e^	990.15 ± 30.04 ^e^	12.47 ± 0.37 ^gh^	3.34 ± 0.01 ^g^	1895.51 ± 34.16 ^e^	16.57 ± 0.36 ^ef^
11	165 (+1)	200 (−1)	3.45 ± 0.02 ^c^	677.37 ± 22.30 ^j^	12.23 ± 0.27 ^h^	3.77 ± 0.01 ^c^	1407.97 ± 39.20 ^j^	16.06 ± 0.34 ^fg^
12	165 (+1)	250 (0)	3.24 ± 0.12 ^e^	729.86 ± 25.92 ^i^	11.54 ± 0.28 ^i^	3.48 ± 0.02 ^f^	1471.48 ± 28.51 ^i^	15.75 ± 0.46 ^fg^
13	165 (+1)	300 (+1)	3.06 ± 0.06 ^f^	800.24 ± 27.08 ^h^	10.72 ± 0.25 ^j^	3.15 ± 0.01 ^h^	1533.62 ± 52.40 ^h^	15.27 ± 0.37 ^g^

*Note:* Coded and actual experimental levels in the central composite design and experimental results for WAI, PA, and ISDF of extruded flours compared to the raw. Factor levels are expressed as actual levels (coded levels). The results presented in the table are on dry basis. The values in the table are the average values of the results obtained in the analyses. *n* = 3 for water absorption index. *n* = 18 for phytic acid and *n* = 2 for insoluble dietary fiber. Data are expressed as mean value ± standard deviation. Mean values within same column with different lowercase superscripts are significantly different (*p* < 0.05). Raw was un‐extrusion cooked flour and it was not part of the design.

Abbreviations: DT, die temperature; ISDF, insoluble dietary fiber; PA, phytic acid; SS, screw speed; WAI, water absorption index.

#### 3.1.1 Water Absorption Index (WAI)

**FIGURE 1:**
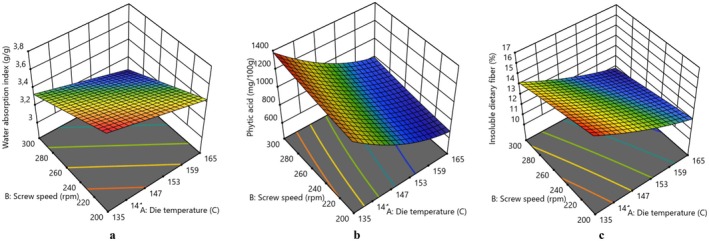
3D surface plots expressing the effect of die temperature and screw speed on (a) water absorption index, (b) phytic acid, (c) insoluble dietary fiber of extruded mung bean flour.

The water absorption index acts as an indicator of starch integrity, reflecting the volume of starch after it undergoes swelling in excess water (Kesselly et al. 2023). As shown in Table 1, all extruded flours exhibited lower WAI values in comparison to their non‐extruded counterparts. The WAI of EMF and EBF was significantly affected by all parameters. The WAI decreases with increasing DT and SS (*p* < 0.05) (Figure 1). The decrease in the WAI was attributed to the harsher effects on starch polymers, resulting in an extensive molecular breakdown in high SS conditions, and consequently causing a decrease in the ability to bind water. In the present study, as the higher DT leads to the degradation or disintegration of starch molecules, the WAI decreases at elevated temperatures, which may result from the melting or dextrinization of the starch at high temperatures. Also, the water absorption of extruded products could be evaluated based on the interactions between starch, water, and protein, governed by the structure of the solid phase. The degree of starch damage, caused by gelatinization and fragmentation induced by extrusion, enhanced starch dispersion in excess water by decreasing the molecular weight of amylose and amylopectin (Alam et al. 2016). As mentioned above, during the extrusion process, the decrease in the molecular size of starch may lead to a lowered capacity of the molecules to bind or retain water, thereby reducing the water absorption index. Additionally, a high residence time (due to the lower SS) in the extruder barrel exposes the starch to high temperatures for a longer period, leading to the degradation or disintegration of the starch molecules. This finding led to the conclusion that the decrease in WAI was attributed to the increased residence time of the product in the extruder barrel, resulting from the decrease in SS. In agreement with our findings, Ek et al. (2021) also found that the WAI was increased as the extrusion temperature and SS decreased in whole seed extruded lentil flour. A similar result has also been reported by Sahu et al. (2022) for soybean‐maize extruded snacks. Ghumman et al. (2016) observed that at lower moisture content (15%), the WAI of lentil extrudates decreased (*p* < 0.05), whereas at higher moisture content (20% and 25%) with increasing extrusion temperature, there was no significant change in the WAI with increasing extrusion temperature. The interplay between the content of moisture and temperature has been demonstrated to affect the WAI of lentil flour (*p* < 0.05) with a positive correlation, whereas the SS reduced the WAI (Rathod and Annapure 2016). Research has shown that extrusion significantly increases the WAI of different pulse extrudates, suggesting that, during the extrusion process, soluble components are released, and starch potentially absorbs more water (Faliarizao et al. 2024).

#### 3.1.2 Phytic Acid (PA) Content

Inositol hexaphosphate (IP6), also referred to as phytic acid or phytate, serves as a primary phosphorus storage compound in mature pulse seeds (Ciudad‐Mulero et al. 2020). Phytate is capable of forming complexes with metal ions, thereby inhibiting the absorption of essential minerals such as iron, zinc, magnesium, and calcium (Samtiya et al. 2020), leading to mineral deficiency in humans and animals. In this study, the PA content of RMF, EMF, RBF, and EBF is presented in Table 1. The RMF and RBF contained 1673 mg/100 g and 2572.60 mg/100 g, respectively. The unprocessed samples exhibit higher PA content than the extruded ones in the current study. Other researchers have reported PA contents as follows: Dahiya et al. (2015) found 737–807 mg/100 g in mung beans; Zafar et al. (2023) reported 450–1220 mg/100 g in mung beans; and Dhole and Reddy (2015) found 574–1898 mg/100 g in summer and 585–2,002 mg/100 g in winter for different mung bean genotypes. The difference between the PA content of pulses can be influenced by genotypes, environmental conditions, types of soil, fertilizer, and location.

**FIGURE 2:**
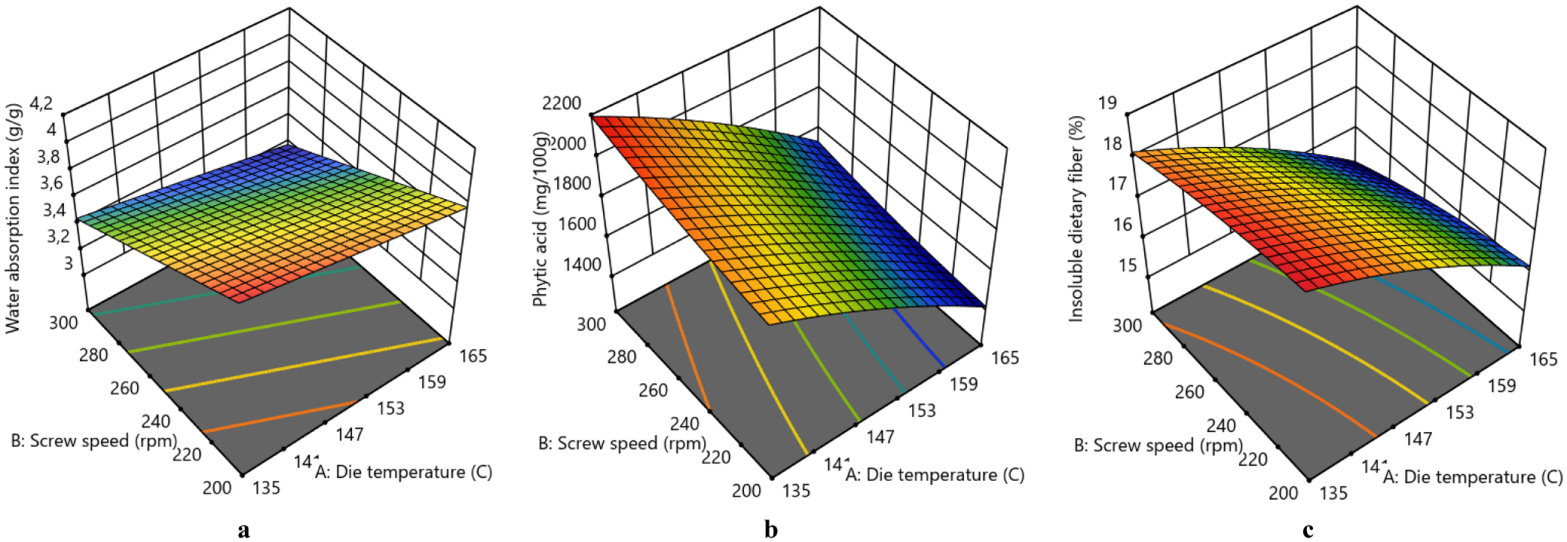
3D surface plots expressing the effect of die temperature and screw speed on (a) Water absorption index, (b) Phytic acid, (c) Insoluble dietary fiber of extruded broad bean flour.

The PA content of flours was significantly affected by both independent variables in the current study. A significant decrease in PA content was observed with increasing DT, whereas a decrease in SS led to the opposite effect (Figures 1 and 2). The reduction in PA content may be attributed to the hydrolysis of PA into lower molecular weight forms owing to the increase in temperature during extrusion cooking. Additionally, the decrease in PA content after extrusion could be partially ascribed to its heat sensitivity. Furthermore, the reduction of PA content may also result from the occurrence of insoluble complexes between phytate and calcium or magnesium during thermal processing (Samtiya et al. 2020). Arribas et al. (2019) also declared that extrusion cooking diminished the IP6 content of extrudates, leading to less phosphorylated forms. The reduction of PA content by extrusion cooking has also been observed by Guillamon et al. (2008) in bean, Rathod and Annapure (2016) in lentil, Yadav et al. (2019) in chickpea and cowpea, Ciudad‐Mulero et al. (2020) in lentil, respectively. In light of this information, it is possible to say that the application of the extrusion process could be an effective processing technology to decrease PA content to a negligible level or lower phosphorylated forms.

#### 3.1.3 Insoluble Dietary Fiber (ISDF)

The ISDF content of raw and extruded flours is presented in Table 1. The ISDF content of RBF exhibited a higher value than RMF. The ISDF content of different types of pulses stated by a previous study, which is 8.64% in mung bean and 14.76% in soybean by Huang et al. (2013), was in the range of 10.70% and 15.29% in different cultivars of broad bean by Labba et al. (2021), and in the range of 13.1% and 19% in mung bean by Dahiya et al. (2015). The ISDF results of RMF and RBF obtained in this study agree with those of other authors.

The ISDF content of both extruded flours decreased with the increasing DT and SS in extrusion (Figures 1 and 2). Higher temperature and relatively lower moisture content used in the extrusion process led to high shear conditions, which caused damage to fiber‐rich cell walls as stated by Espinosa‐Ramírez et al. (2021).

It is possible to say that the moisture and heat conditions used in extrusion can solubilize and degrade the pectic compounds and therefore reduce dietary fiber (Arribas et al. 2019). Extrusion is achieved to break down polysaccharide bonds and to rupture the macro and microstructure of the fiber matrix, leading to ISDF degradation (Berrios 2011). The higher SS reduces the residence time in the barrel, but the product is exposed to higher shear stress and friction. This may contribute to the observed reduction in ISDF content of the flour after extrusion. In agreement with our study, Alam et al. (2016) observed that the SS and temperature conducted in the extrusion process have presented a profound effect on the fiber content of the product. A significant reduction in ISDF for extruded formulations based on lentil‐based by Morales et al. (2015); for rice‐based by Arribas et al. (2019); and for oat, sorghum, amaranth, quinoa, chickpea, lentil, black bean, and pinto bean flours by Espinosa‐Ramírez et al. (2021) (*p* < 0.05).

#### 3.1.4 Optimization and Verification of Results

In the current study, the WAI, PA, and ISDF were chosen as the response variables for optimization. The optimal solution was obtained through software‐based analysis designed to simultaneously maximize WAI and minimize PA and ISDF levels. The predictive model for optimal responses was experimentally validated by producing an additional batch. The response analyses were also repeated. The experimental and predicted values of the responses at optimum conditions are presented in Table 2. The optimum conditions were determined to be the same as both flour types were 165°C DT and 200 rpm SS, resulting in a desirability value of 0.763 for EMF and 0.800 for EBF, respectively. The predicted values of the responses are within a 95% confidence interval, confirming that the selected models accurately predict the experimental results. The experimentally obtained response values under optimal conditions were consistent with the predicted values.

**TABLE 2:** Predicted and experimental values of the responses at optimum conditions 165°C die temperature and 200 rpm screw speed.

Responses	95% PI low	Experimental results	95% PI high
	**Mung bean**		
WAI (g/100 g)	3.3863	3.46384	3.50389
PA (g/100 g)	662.874	676.48	697.266
ISDF (%)	11.7379	12.0458	12.5809
	**Broad bean**		
WAI (g/100 g)	3.71987	3.75726	3.84924
PA (g/100 g)	1389.24	1405.77	1442.78
ISDF (%)	15.5856	16.0794	16.7732

Abbreviations: ISDF, insoluble dietary fiber; PA, phytic acid; WAI, water absorption index.

### 3.2 Properties of Flour and Flour Blends

The blends were prepared by replacing WF with RMF, RBF, EMF, and EBF at 12.5% and 25% (w/w), respectively. WF was used as the control sample. The extruded flours used in this study were produced at optimum extrusion conditions.

The proximate properties of flours used in this study are presented in Table S2. The water absorption capacity values of WF and flour blends were found as follows: 58.5% for WF, 56.1% for 12.5% RMF containing flour blend, 55.2% for 25% RMF containing flour blend, 55.3% for 12.5% EMF containing flour blend, 53.8% for 25% EMF containing flour blend, 55.4% for 12.5% RBF containing flour blend, 53.8% for 25% RBF containing flour blend, 56.3% for 12.5% EBF containing flour blend, and 55.7% for 25% EBF containing flour blends.

#### 3.2.1 Pasting Properties

**FIGURE 3:**
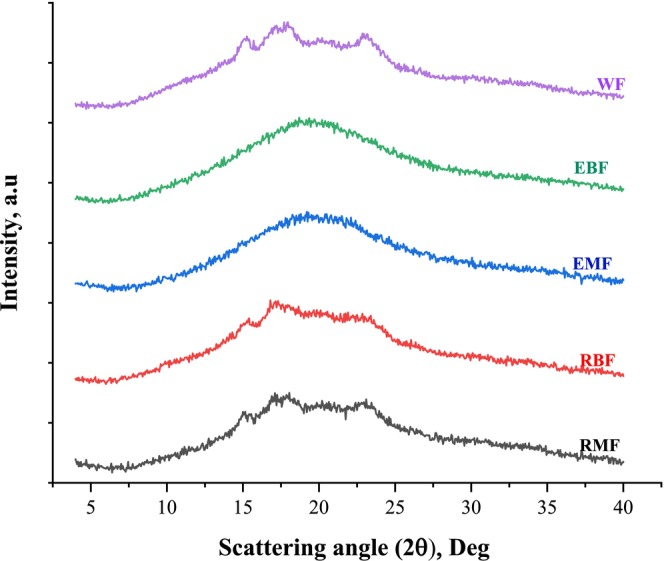
The X‐ray diffraction (XRD) spectra of the flour samples. EBF, extruded broad bean flour; EMF, extruded mung bean flour; RBF, raw broad bean flour; RMF, raw mung bean flour; WF, wheat flour.

The pasting properties of WF extruded and non‐extruded mung bean and broad bean flours, and their blends were examined, and the pasting temperature, IPV/peak viscosity (PV), hot paste viscosity (HPV), and final viscosity (cold paste viscosity, CPV) of the samples were determined (Figure 3, Table 3). Pasting temperature, the temperature at which the product's viscosity begins to increase during the process of heating, may be interpreted as an indicator of the resistance of the starch material in the product against gelatinization. Peak viscosity value, or the greatest value of viscosity measured during the phase of heating, was registered when the level of viscosity reached 90°C. For flours that were extruded, the PV value was determined by the maximum viscosity displayed by the paste in the peak generated by combining the inflection points of the baseline viscosity curves during the heating phase (Guha et al. 1998). The decrease in viscosity after thinning (breakdown viscosity‐ BDV) was calculated as the difference between PV and HPV.

**TABLE 3:** Pasting properties of flours.

Sample	Pasting temperature (PaT, °C)	Peak viscosity/Initial peak viscosity (PV/IPV, cP)	Hot paste viscosity (HPV, cP)	Final viscosity/Cold paste viscosity (cP)	Breakdown viscosity (BDV, cP)
WF	67.76 ± 0.25^cd^	575.60 ± 13.44^h^	302.69 ± 5.17^f^	1091.91 ± 16.31^h^	272.91 ± 8.27^e^
RMF	68.67 ± 0.58^d^	281.75 ± 26.57^d^	—	652.02 ± 66.85^e^	—
RBF	67.29 ± 3.07^cd^	117.29 ± 3.84^b^	—	324.71 ± 9.35^b^	—
EMF	—	71.18 ± 2.55^a^	35.49 ± 4.12^a^	92.29 ± 11.13^a^	35.69 ± 6.67^a^
EBF	—	70.35 ± 7.31^a^	44.74 ± 1.11^a^	116.20 ± 3.80^a^	25.61 ± 6.20^a^
12.5‐RMF	62.45 ± 0.04^a^	543.57 ± 18.84^g^	282.63 ± 9.91^e^	937.69 ± 33.12^g^	260.94 ± 8.92^e^
25‐RMF	62.38 ± 0.09^a^	585.34 ± 9.66^h^	308.47 ± 0.81^f^	943.68 ± 6.98^g^	276.87 ± 8.85^e^
12.5‐EMF	66.45 ± 0.17^cd^	363.17 ± 4.86^e^	199.81 ± 5.22^c^	664.70 ± 18.07^e^	163.35 ± 0.36^c^
25‐EMF	66.00 ± 0.51^cd^	235.24 ± 4.11^c^	142.56 ± 5.41^b^	431.21 ± 25.60^c^	92.69 ± 1.30^b^
12.5‐RBF	65.44 ± 0.05^bc^	531.21 ± 12.65^g^	298.55 ± 3.49^ef^	978.15 ± 22.72^g^	232.66 ± 9.16^d^
25‐RBF	63.15 ± 0.47^ab^	525.21 ± 12.78^g^	349.75 ± 20.29^g^	967.32 ± 17.95^g^	175.46 ± 33.07^c^
12.5‐EBF	67.57 ± 0.02^cd^	407.17 ± 2.93^f^	227.18 ± 6.41^d^	744.90 ± 23.14^f^	179.99 ± 3.49^c^
25‐EBF	66.67 ± 2.37^cd^	282.91 ± 9.88^d^	183.21 ± 5.24^c^	523.26 ± 2.23^d^	99.70 ± 15.12^b^

*Note:* Data are expressed as mean value ± standard deviation. *n* = 2.

Abbreviations: 12.5‐EBF, 12.5% extruded broad bean flour‐87.5% wheat flour blend; 12.5‐EMF, 12.5% extruded mung bean flour‐87.5% wheat flour blend; 12.5‐RBF, 12.5% raw broad bean flour‐87.5% wheat flour blend; 12.5‐RMF, 12.5% raw mung bean flour‐87.5% wheat flour blend; 25‐EBF, 25% extruded broad bean flour‐75% wheat flour blend; 25‐EMF, 25% extruded mung bean flour‐75% wheat flour blend; 25‐RBF, 25% raw broad bean flour‐75% wheat flour blend; 25‐RMF, 25% raw mung bean flour‐75% wheat flour blend; EBF, extruded broad bean flour; EMF, extruded mung bean flour; nd, not detected; RBF, raw broad bean flour; RMF, raw mung bean flour; WF, wheat flour.

Non‐extruded and extruded pulse flours and their blends showed distinctive patterns as a function of botanical source and processing in pasting characteristics. WF showed a Type A pasting curve (Schoch and Maywald 1968) with high PV, final viscosity, and large breakdown viscosity, which indicated rapid granule swelling and shear‐thinning with heat. Conversely, non‐extruded pulse flours had Type C curves, with considerably lower PV and no detectable BDV, with low granule swelling, which points to high hot‐paste stability (Abbas et al. 1986). Extrusion resulted in permanent structural changes in legume flours and shifted these profiles to Type D curves, which are marked by minimal viscosity development due to starch granule fragmentation (Abbas et al. 1986). IPV was lower in extruded samples, and CPV was negligible. The occurrence of minimal BDV in extruded flours revealed partial resistance of fragmented starch to shear. This suppression of retrogradation in extruded samples indicated extensive mechanical degradation of starch structure and disrupted amylose‐amylopectin bonds, as seen through extremely low final viscosity.

Non‐extruded blends had a viscosity comparable to WF. This preservation would have been brought about by the presence of undamaged starch granules and synergistic protein‐starch interactions, which stabilized the matrix under heat. Nevertheless, extruded blends had irreversible damage to starch because of mechanical shear and heat degradation. The addition of pulse flour to the blend reduced the pasting temperature. This decrease in pasting temperature was also most significant when extruded mung bean was added to the formula and may be attributed to starch granule breakup. With the level of BB and MB flours in the formula increased, PV values were decreased. This decrease in PV is thought to occur since the high proportion of protein contained in the flours competes with the starch for the water employed in gelatinization of the starch, thereby hindering the gelatinization of the starch (Wang et al. 2020). Additionally, the decrease in PV was most significant in samples formulated with extruded legume flours, which might be attributed to the starch damage. BDV is used to evaluate how much the starch granules break down during continuous shearing at high temperatures. The addition of pulse flours to the formulation led to a decrease in BDV, implying stabilized granule integrity via protein‐starch interactions.

The final viscosity is indicative of the structure formed on cooling by its break resistance. WF possesses the maximum final viscosity with the strong retrogradation tendency characteristic of it. It results from the reorientation of leached amylose chains in cooling that get reassociated as a strong, rigid crystalline network, being typical for Type A starch systems (Schoch and Maywald 1968). The addition of pulse flours led to a decrease in final viscosity values, which might be due to the high protein content of legume flours that can competitively bind water, thereby limiting starch hydration and recrystallization. Extruded pulse flours showed drastically reduced final viscosity values. This suppression of retrogradation can be attributed to the mechanical and thermal degradation of starch during extrusion, which fragments amylopectin into short linear glucans and disrupts the ordered amylose‐amylopectin interactions necessary for recrystallization. As the amount of extruded legume flours in the formulation increased, the final viscosity values decreased, which might be attributed to starch damage.

#### 3.2.2 X‐Ray Diffraction (XRD)

Figure 3 illustrates the XRD pattern of the bread making flour samples. The WF exhibited peaks at 15.25°, 17.95°, 22.85°, and 26.65° (2θ), reflecting A‐type diffraction (Chen et al. 2019). Cereal grains contain A‐type starches, which have varying structural and functional properties.

The RMF showed peaks of characteristic nature at 5.53°, 15.15°, 17.1°, 18.05°, 20.02°, and 23.1° (2θ). The XRD pattern of RBF showed intense peaks with a shoulder peak at 15.25°, 17.95°, 20.02°, and 22.85° (2θ). These peaks indicate that RMF and RBF have C‐type XRD patterns, a mixture of A‐ and B‐type polymorphs in varying proportions (Li et al. 2019). The C‐type starches are predominantly found in legume starches and are often associated with relatively higher amylose content, longer amylopectin branch chain length, and closer bonding, which results in their gelatinization temperature and enthalpy being higher (Bangar et al. 2022). Their structural characteristics provide C‐type starches with special crystal adaptability and are the reason for their lower digestibility (Guo et al. 2017).

After extrusion, all the characteristic peaks in the XRD patterns of mung bean and broad bean flours disappeared or were drastically reduced, confirming that extrusion disrupts the crystalline structure of starch and transforms it into a largely amorphous state. The disappearance of these peaks confirms that the ordered starch granules were ruptured by the combined effects of high shear, pressure, and temperature. The mechanism of an amorphous structure transformation during gelatinization increases the susceptibility of starch to enzymatic hydrolysis and water absorption (Wang and Copeland 2013) which renders the extruded flours more suitable for incorporation in bread formulations.

#### 3.2.3 FTIR

**FIGURE 4:**
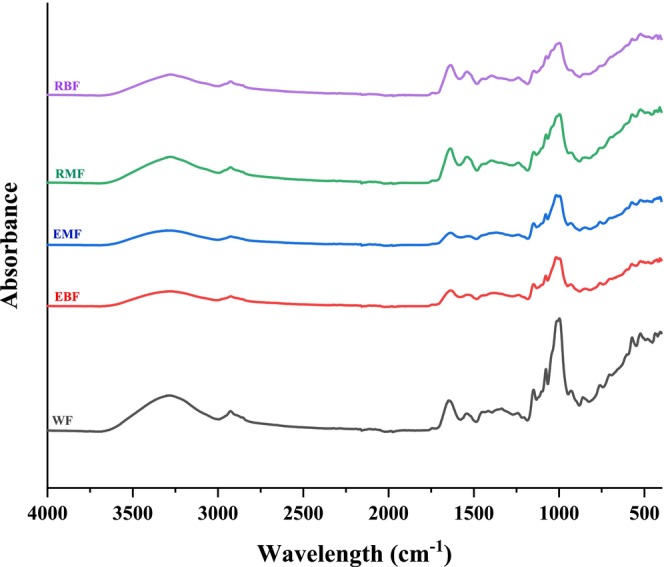
The FTIR spectra of flours. EBF, extruded broad bean flour; EMF, extruded mung bean flour; RBF, raw broad bean flour; RMF, Raw mung bean flour; WF, wheat flour.

FTIR spectra of RMF, RBF, WF, EMF, and EBF are provided in Figure 4. Spectra indicate characteristic bands of absorption by the functional groups of lipids, proteins, and carbohydrates. The broadband at 3279 cm^−1^ corresponds to O‐H stretching vibrations, primarily by hydroxyl groups of polysaccharides and proteins. The band at 2926 cm^−1^ is a peak for absorption due to the asymmetric stretch of C‐H bonds in CH_2_ groups that are commonly related to protein and lipid structures (Gordon et al. 2019). Similarly, this region also associated with Amide B vibrations such as $-{\mathrm{NH}}_{3}^{+}$ and =C–H stretching.

The characteristic Amide B, Amide I, and Amide II peaks, associated with protein secondary structures, were identified at 2926, 1635, and 1538 cm^−1^, respectively, in mung bean and broad bean flours, whereas these bands were observed at 2926, 1645, and 1541 cm^−1^ in WF. The amide B peaks signify the asymmetric stretch vibration of =C–H as well as $-{\mathrm{NH}}_{3}^{+}$ (Ahmad et al. 2010). Amide I primarily arises from C=O stretching vibrations in peptide bonds, whereas Amide II is attributed to N‐H bending and C‐N stretching (Johnson et al. 2020). Extrusion processing decreased the intensity of Amide B, Amide I, and II bands that indicate protein denaturation and structural modifications that could be attributed to the disruption of hydrogen bonds and changes in protein secondary structures that cause partial unfolding or aggregation (Pavani et al. 2024).

The 1480–1184 cm^−1^ spectral range is often associated with carbohydrate vibrations, whereas the bands in the 1184–875 cm^−1^ region are also related to carbohydrates(Zhang and Yu 2012). Extrusion led to shifts in these ranges due to starch gelatinization and carbohydrate structure breakdown. The presence of new bands or variations in this region signals changes in glycosidic linkages and possible depolymerization of polysaccharides.

The changes observed in the FTIR spectra correspond to the extrusion effects on the molecular organization of legume flours that would influence their functional properties, including water absorption, digestibility, and textural properties.

#### 3.2.4 Thermal Properties

Thermal characteristics of WF, RMF, RBF, EMF, and EBF were examined using DSC. The peak temperature of this stage was taken as the gelatinization temperature. The onset temperature and peak temperature of WF were 57.67°C and 65.47°C, respectively. For RMF, the onset of gelatinization was observed at 82.60°C, and its peak was at 83.31°C; whereas the onset temperature of RBF was recorded at 76.45°C with a peak at 76.67°C. Similar results were reported in research conducted with WF, mung bean, and broad bean flour. Fujita et al. (1992) determined that the gelatinization temperature for WF is around 57°C; whereas Sosulski et al. (1985) determined that pulse flours like mung bean and broad bean flours require considerably higher temperatures, usually ranging from 76°C to 83°C. This shows the difference in gelatinization behavior among species, where significantly higher temperatures and enthalpy are required by pulse flours in comparison to WF (Fujita et al. 1992; Sosulski et al. 1985). No detectable gelatinization occurred in extruded pulse flours, which suggests that legume starches should already have been gelatinized by extrusion due to the high pressure and heat used.

#### 3.2.5 Dynamic Oscillation Characteristics

**FIGURE 5:**
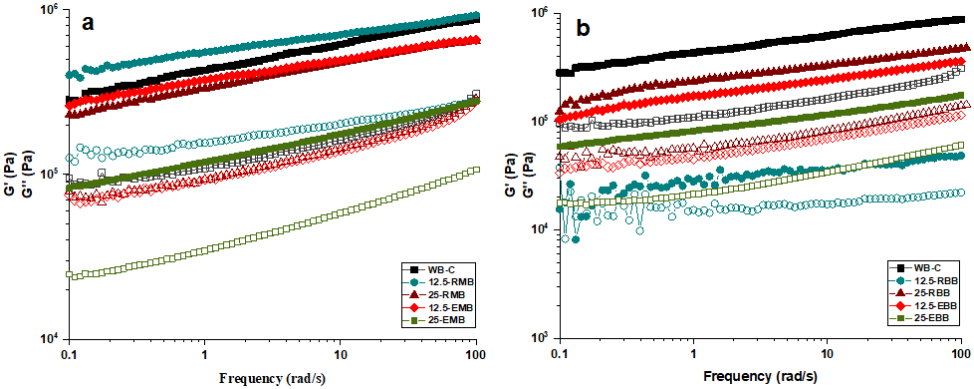
Storage modulus (G′, solid markers) and loss modulus (G″, open markers) of samples during frequency sweep tests. Different marker shapes and colors correspond to the sample formulations indicated in the legend. 12.5‐EBB, bread dough prepared with 12.5% extruded broad bean flour‐87.5% wheat flour; 12.5‐EMB, bread dough prepared with 12.5% extruded mung bean flour‐87.5% wheat flour; 12.5‐RBB, bread dough prepared with 12.5% raw broad bean flour‐87.5% wheat flour; 12.5‐RMB, bread dough prepared with 12.5% raw mung bean flour‐87.5% wheat flour; 25‐EBB, bread dough prepared with 25% extruded broad bean flour‐75% wheat flour; 25‐EMB, bread dough prepared with 25% extruded mung bean flour‐75% wheat flour; 25‐RBB, bread dough prepared with 25% raw broad bean flour‐75% wheat flour; 25‐RMB, bread dough prepared with 25% raw mung bean flour‐75% wheat flour; WB‐C, bread dough prepared with wheat flour (control).

Loss modulus (*G*″) and storage modulus (*G*′) of dough systems prepared from various ratios of RMF, RBF, EMF, and EBF were determined as a function of frequency, and results are presented in Figure 5. In all the formulations, values of *G*′ were higher than values of *G*″, indicating solid‐like behavior of the dough systems. Both moduli increased with rising frequency, reflecting the frequency‐dependent strengthening of the dough matrix. With the exception of the 12.5‐RMF sample, the WF sample exhibited maximum *G*′ and *G*″, which reflects greater elastic and viscous responses compared to legume‐incorporated doughs. The incorporation of raw and extruded mung bean and broad bean flours caused a significant reduction in both *G*′ and *G*″, which reflects a loss of strength in the dough network. This decrease can be attributed to the dilution effect caused by the partial replacement of wheat gluten with legume proteins (Olakanmi et al. 2022), which inherently have lower elasticity and create a less cohesive dough structure. In the comparison of samples with raw and extruded pulse flour, the latter reduced the values of G′ and G′′ further. The results suggest that starch gelatinization and protein denaturation, occurring in the pulse flour during extrusion, alter the hydration properties and viscoelasticity of the dough. The substitution of pulse flours at 25% decreased the *G*′ and *G*″ significantly compared to the substitution of pulse flours at 12.5%, which suggests that increasing the level of pulse flours further disrupts the gluten network more, leading to a weaker, less cohesive network with reduced resistance to deformation under stress.

### 3.3 Bread Properties

In bread making trials, WF was substituted with RMF, RBF, EMF, and EBF at 12.5% and 25% (w/w), respectively. WF bread was also produced as the control bread (WB‐C).

#### 3.3.1 Quality Characteristics of Breads

In this study, quality characteristics of bread samples are comprised of: loaf volume, specific volume, baking loss, moisture content, color values (L*, a*, b*), and texture profile analysis (hardness, springiness, cohesiveness, chewiness, resilience).

**FIGURE 6:**
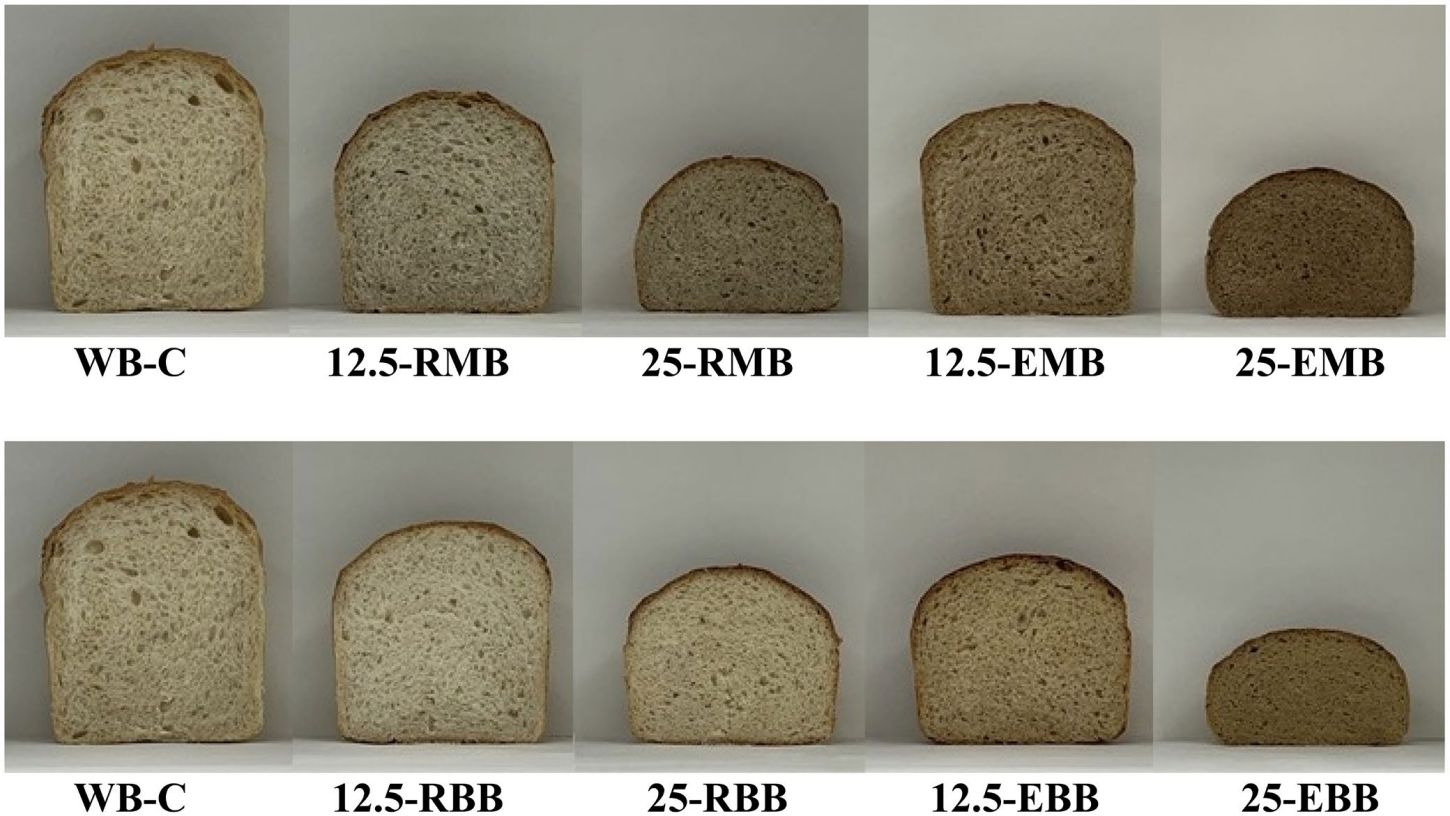
Cross‐section views of bread samples. 12.5‐EBB, 12.5% extruded broad bean flour‐87.5% wheat flour bread; 12.5‐EMB, 12.5% extruded mung bean flour‐87.5% wheat flour bread; 12.5‐RBB, 12.5% raw broad bean flour‐87.5% wheat flour bread; 12.5‐RMB, 12.5% raw mung bean flour‐87.5% wheat flour bread; 25‐EBB, 25% extruded broad bean flour‐75% wheat flour bread; 25‐EMB, 25% extruded mung bean flour‐75% wheat flour bread; 25‐RBB, 25% raw broad bean flour‐75% wheat flour bread; 25‐RMB, 25% raw mung bean flour‐75% wheat flour bread; WB‐C, wheat flour bread (control bread).

Loaf volume is a key indicator of bread quality. It is well‐known that when WF is replaced with non‐WF, the volume of bread decreases depending on the substitution level. In our study, the highest specific volume was found in the WB‐C among the bread samples, as we expected (Table 4). The increasing substitution levels of RMF, EMF, RBF, and EBF from 12.5% to 25% caused a notable decrease in the specific volume of bread, compared to the WB‐C (*p* < 0.05) (Figure 6). It is well‐known that during the kneading stage in bread making, gluten proteins, which are comprised of gliadins and glutenins, are hydrated by water, and form the gluten network. Gluten proteins, especially glutenins, play a key role in dough extensibility, gas retention, and bread volume (Batista et al. 2011).

**TABLE 4:** Quality characteristics of bread samples.

Bread samples	Loaf volume (cm^3^)	Specific volume (cm^3^/g)	Baking loss (%)	Moisture content (%)	L*	a*	b*
WB‐C	772.55 ± 25.09^a^	5.15 ± 0.18^a^	14.37 ± 0.44^a^	34.27 ± 0.53^c^	77.99 ± 1.96^a^	0.03 ± 0.01^e^	12.58 ± 0.72^e^
12.5‐RMB	636.00 ± 21.46^b^	4.22 ± 0.15^b^	13.95 ± 0.42^b^	35.81 ± 0.31^b^	76.13 ± 0.22^b^	0.62 ± 0.10^d^	13.48 ± 0.71^d^
25‐RMB	405.95 ± 15.99^d^	2.62 ± 0.10^d^	11.69 ± 0.32^c^	36.66 ± 0.27^a^	68.23 ± 1.40^d^	1.34 ± 0.11^c^	17.20 ± 0.55^c^
12.5‐EMB	617.77 ± 15.27^c^	4.09 ± 0.11^c^	13.85 ± 0.24^b^	35.59 ± 0.38^b^	71.74 ± 2.63^c^	3.23 ± 0.36^b^	18.73 ± 1.05^b^
25‐EMB	366.00 ± 15.90^e^	2.32 ± 0.11^e^	10.11 ± 0.47^d^	36.63 ± 0.44^a^	57.92 ± 1.58^e^	5.59 ± 0.36^a^	20.15 ± 1.05^a^
WB‐C	772.55 ± 25.09^A^	5.15 ± 0.18^A^	14.37 ± 0.44^A^	34.27 ± 0.53^C^	77.99 ± 1.96^A^	0.03 ± 0.01^E^	12.58 ± 0.72^D^
12.5‐RBB	672.37 ± 14.08^B^	4.46 ± 0.10^B^	13.87 ± 0.31^B^	35.68 ± 0.30^B^	76.87 ± 1.81^B^	0.26 ± 0.05^D^	12.99 ± 0.67^D^
25‐RBB	459.31 ± 23.40^D^	2.96 ± 0.14^D^	11.60 ± 0.36^D^	36.32 ± 0.38^A^	75.63 ± 1.33^C^	0.71 ± 0.10^C^	17.86 ± 0.67^C^
12.5‐EBB	515.39 ± 11.34^C^	3.40 ± 0.08^C^	13.44 ± 0.47^C^	35.56 ± 0.17^B^	73.49 ± 2.01^D^	2.38 ± 0.29^B^	20.93 ± 0.96^B^
25‐EBB	273.50 ± 2.89^E^	1.72 ± 0.02^E^	9.38 ± 0.36^E^	36.39 ± 0.33^A^	66.91 ± 1.58^E^	5.24 ± 0.34^A^	25.38 ± 1.07^A^

*Note:* Data are expressed as mean value ± standard deviation. Mean values within the same column with different lowercase and uppercase superscripts are significantly different (*p* < 0.05). *n* = 12 for loaf volume, specific volume, and baking loss; *n* = 8 for moisture content; *n* = 24 for color (L*, a*, b*) values.

Abbreviations: 12.5‐EBB, 12.5% extruded broad bean flour‐87.5% wheat flour bread; 12.5‐EMB, 12.5% extruded mung bean flour‐87.5% wheat flour bread; 12.5‐RBB, 12.5% raw broad bean flour‐87.5% wheat flour bread; 12.5‐RMB, 12.5% raw mung bean flour‐87.5% wheat flour bread; 25‐EBB, 25% extruded broad bean flour‐75% wheat flour bread; 25‐EMB, 25% extruded mung bean flour‐75% wheat flour bread; 25‐RBB, 25% raw broad bean flour‐75% wheat flour bread; 25‐RMB, 25% raw mung bean flour‐75% wheat flour bread; WB‐C, wheat flour bread (control bread).

In our study, the decrease in specific volume was linked to the weakened gluten network formation in dough from flour blends, leading to a reduced rise during proofing due to the weaker gluten structure. Since broad bean and mung bean do not contain gluten proteins, the substitution of WF with MBF and BBF caused a decrease in the ratio of gluten proteins in the total mass of flour blends depending upon the substitution levels, and this resulted in a lower loaf volume in bread produced from flour blends. On the other hand, the gluten‐starch matrix most probably affected the volume of bread. Since the main constituents of WF are starch and protein (Gao et al. 2020), starch also plays a multifunctional role in the viscoelastic behavior of WF dough (Hu et al. 2023). The relationships between gluten network and starch granules influence dough development, and gluten‐starch interactions determine the rheological properties dough (Gao et al. 2020). Sroan et al. (2009) reported that the gluten‐starch matrix stabilizes the gas cell membrane to ensure dough expansion during proofing and baking. In our opinion, since gluten‐starch interactions play an important role in stabilizing the enlarging gas cells during proofing and baking, the decrease in the degree of gluten‐starch matrix form in dough prepared from flour blends exhibited a detrimental effect on the loaf volume of bread samples in our study. Similar to our findings on the specific volume of bread samples, Wang et al. (2018) observed that incorporating 30% broad bean flour into WF resulted in a notable reduction in the specific volume of the bread relative to that made with 100% WF.

In another study by Coda et al. (2017), it was reported no significant difference in the specific volume between bread made solely from WF and that produced using a flour blend (70% WF:30% broad bean flour), the specific volume of bread produced from flour blend was found to be lower than that of the WF bread. According to our results, we are of the opinion that another factor that affected and decreased the loaf volume was the fiber content in the flour blends. The higher amount of total dietary fiber in RMF, EMF, RBF, and EBF caused an increase in the total dietary fiber contents in flour blends, depending upon the substitution levels, which resulted in a notable decrease in the loaf volume produced from flour blends. Akın et al. (2021), who used tea fiber at 2.5%–5%–7.5%–10% levels in WF bread production, found that the higher fiber content caused a significant decrease in loaf volume.

Considering the loaf volume between RMF, EMF, RBF, and EBF, respectively, we state that the specific volume values of breads that contain RMF and RBF are higher than those of EMF and EBF containing bread samples (*p* < 0.05). This finding may be attributed to the gelatinization of starch granules in extruded flours. In our study, since we used EMF and EBF in which starch has already been gelatinized in the extrusion process, the amount of un‐gelatinized starch in the total mass of the flour blends was reduced. Thereby, sufficient expansion of gas cells was not exhibited; concomitantly, the volume of breads that contained EMF and EBF was found to be lower than RMF and RBF containing bread samples. It was reported that starch pasting properties play an important role in dough expansion. It is suggested that starch gelatinization during heating contributes to the solidification of the gas cell membranes in dough. Therefore, early onset of starch granule gelatinization should be avoided to allow sufficient time for gas cell expansion during the baking process (Hu et al. 2023). Regarding the baking loss values of bread samples, we observed that WB‐C had the highest baking loss among the brad samples (*p* < 0.05). The increasing substitution levels of RMF, EMF, RBF, and EBF caused a significant decrease in baking loss compared to WB‐C (*p* < 0.05). This decrease in the baking loss was attributed to the water absorption capacity of the WF and the flour blends. The decrease in the water absorption capacity in flour blends resulted in a decrease in the baking loss of bread samples. This finding was attributed to the higher free water in WF dough arises from the higher water absorption capacity of WF. In our opinion, the higher amount of free water in the dough removed during the baking, consequently, the baking loss value increased. Besides, a decreased level of free water in the dough resulted in a reduction in the baking loss value (Table 4). In agreement with our results, Menon et al. (2015), who produced breads from flour blends that contained different ratios of WF, soy flour, sprouted mung bean flour, and mango kernel flour, found that WF bread had the highest baking loss among the bread samples.

According to the moisture contents of bread samples, we state that the lowest moisture content was found in the WB‐C (*p* < 0.05). The moisture contents of RMF and RBF containing breads were found to be lower than those of EMF and EBF containing breads. These findings were attributed to the baking loss of the bread samples. The higher baking loss in bread exhibited the lower moisture content (Table 4).

The crumb of the WB‐C had the highest L* value and the lowest a* and b* values among the bread samples (*p* < 0.05). The increasing substitution levels of RMF, EMF, RBF, and EBF from 12.5% to 25% caused a significant increase in the L* value and caused a significant decrease in the a* and b* values in bread crumb (*p* < 0.05). Besides, the L* values of the crumbs of the bread samples that contained RMF and RBF were found to be higher than EMF and EBF containing bread samples (*p* < 0.05). We also observed that the a* and b* values of the crumbs of the bread samples containing RMF and RBF were found to be lower than EMF and EBF containing bread samples (*p* < 0.05). Regarding these findings, we conclude that the changes in the color of the crumbs of bread samples depended upon the different L*, a*, and b* values of WF and flour blends, which were presented in Table S2.

As seen in Table 5, the crumb structure of the WB‐C had the lowest hardness value among the bread samples produced from flour blends (*p* < 0.05). This finding led to the conclusion that WB‐C had a softer bread crumb structure among the bread samples produced from flour blends. This finding was attributed to the reduced gluten network in flour blends. The formation of soft crumb structures in bread is largely attributed to the functionality of gluten proteins. The 3‐dimensional gluten network stabilizes the gas (CO_2_) produced throughout the fermentation stages and improves the crumb structure of bread (Batista et al. 2011). We inferred that bread crumb density, which is a major determinant of bread crumb structure, is strongly associated with the volume of bread, and it is related to gluten network properties. A higher loaf volume exhibits a lower crumb density. In our study, we found that the WB‐C showed the lowest crumb hardness (*p* < 0.05) due to the higher loaf volume, and concomitantly lower crumb density. We observed that the increasing substitution levels of RMF, EMF, RBF, and EBF caused a significant increase in the hardness of the bread crumb in comparison with WB‐C (*p* < 0.05). These findings were attributed to the lowered gluten network formation in dough with the increasing substitution levels of mung bean flour and broad bean flour. On the other hand, the bread samples produced from RMF and RBF containing blends exhibited a softer crumb structure compared to the bread samples produced from EMF and EBF containing blends (*p* < 0.05). Considering this finding, we are of the opinion that the gelatinized starch in the EMF and EBF caused a detrimental effect on the crumb hardness of bread. As aforementioned above, the interactions between gluten and starch determine the rheological behavior of dough and its capacity to retain gas (CO_2_).

**TABLE 5:** Texture profile analysis of bread samples.

Bread samples	Hardness (g)	Springiness	Cohesiveness	Chewiness (g)	Resilience
WB‐C	91.61 ± 6.63^d^	0.47 ± 0.02^a^	0.37 ± 0.02^d^	15.82 ± 1.11^e^	0.15 ± 0.01^e^
12.5‐RMB	179.77 ± 12.28^c^	0.44 ± 0.02^b^	0.48 ± 0.02^a^	39.64 ± 2.52^c^	0.20 ± 0.01^a^
25‐RMB	758.89 ± 44.16^b^	0.44 ± 0.01^b^	0.47 ± 0.01^a^	158.44 ± 9.38^a^	0.19 ± 0.01^b^
12.5‐EMB	176.44 ± 12.05^c^	0.34 ± 0.02^d^	0.46 ± 0.02^b^	24.76 ± 1.74^d^	0.17 ± 0.01^d^
25‐EMB	916.78 ± 51.36^a^	0.42 ± 0.02^c^	0.40 ± 0.02^c^	136.68 ± 0.08^b^	0.17 ± 0.01^c^
WB‐C	91.61 ± 6.63^E^	0.47 ± 0.02^A^	0.37 ± 0.02^B^	15.82 ± 1.11^E^	0.15 ± 0.01^D^
12.5‐RBB	150.42 ± 10.35^D^	0.42 ± 0.02^C^	0.47 ± 0.02^A^	31.22 ± 2.06^D^	0.19 ± 0.01^A^
25‐RBB	545.40 ± 35.70^B^	0.44 ± 0.02^B^	0.46 ± 0.02^A^	105.43 ± 7.35^B^	0.19 ± 0.01^B^
12.5‐EBB	331.18 ± 19.25^C^	0.30 ± 0.01^D^	0.37 ± 0.01^B^	36.43 ± 2.14^C^	0.16 ± 0.01^C^
25‐EBB	1718.81 ± 51.23^A^	0.31 ± 0.01^D^	0.34 ± 0.01^C^	178.89 ± 5.59^A^	0.14 ± 0.01^E^

*Note:* Data are expressed as mean value ± standard deviation. Mean values within same column with different lowercase and uppercase superscripts are significantly different (*p* < 0.05). *n* = 16.

Abbreviations: 12.5‐EBB, 12.5% extruded broad bean flour‐87.5% wheat flour bread; 12.5‐EMB, 12.5% extruded mung bean flour‐87.5% wheat flour bread; 12.5‐RBB, 12.5% raw broad bean flour‐87.5% wheat flour bread; 12.5‐RMB, 12.5% raw mung bean flour‐87.5% wheat flour bread; 25‐EBB, 25% extruded broad bean flour‐75% wheat flour bread; 25‐EMB, 25% extruded mung bean flour‐75% wheat flour bread; 25‐RBB, 25% raw broad bean flour‐75% wheat flour bread; 25‐RMB, 25% raw mung bean flour‐75% wheat flour bread; WB‐C, wheat flour bread (control bread).

Given the critical role of starch in determining bread quality, the gelatinized starch in the EMF and EBF decreased the loaf volume, concomitantly increased the bread crumb density, and increased the crumb hardness of the bread.

Considering the chewiness of the bread crumb, we observed that the WB‐C had the lowest chewiness value among the bread samples produced from flour blends (*p* < 0.05). The increasing substitution levels of RMF, EMF, RBF, and EBF caused a notable increase in the chewiness of the bread crumb in comparison with WB‐C (*p* < 0.05). In our study, no gradual changes were observed in the springiness, cohesiveness, and resilience values among the crumb structure of bread samples.

#### 3.3.2 SEM

**FIGURE 7:**
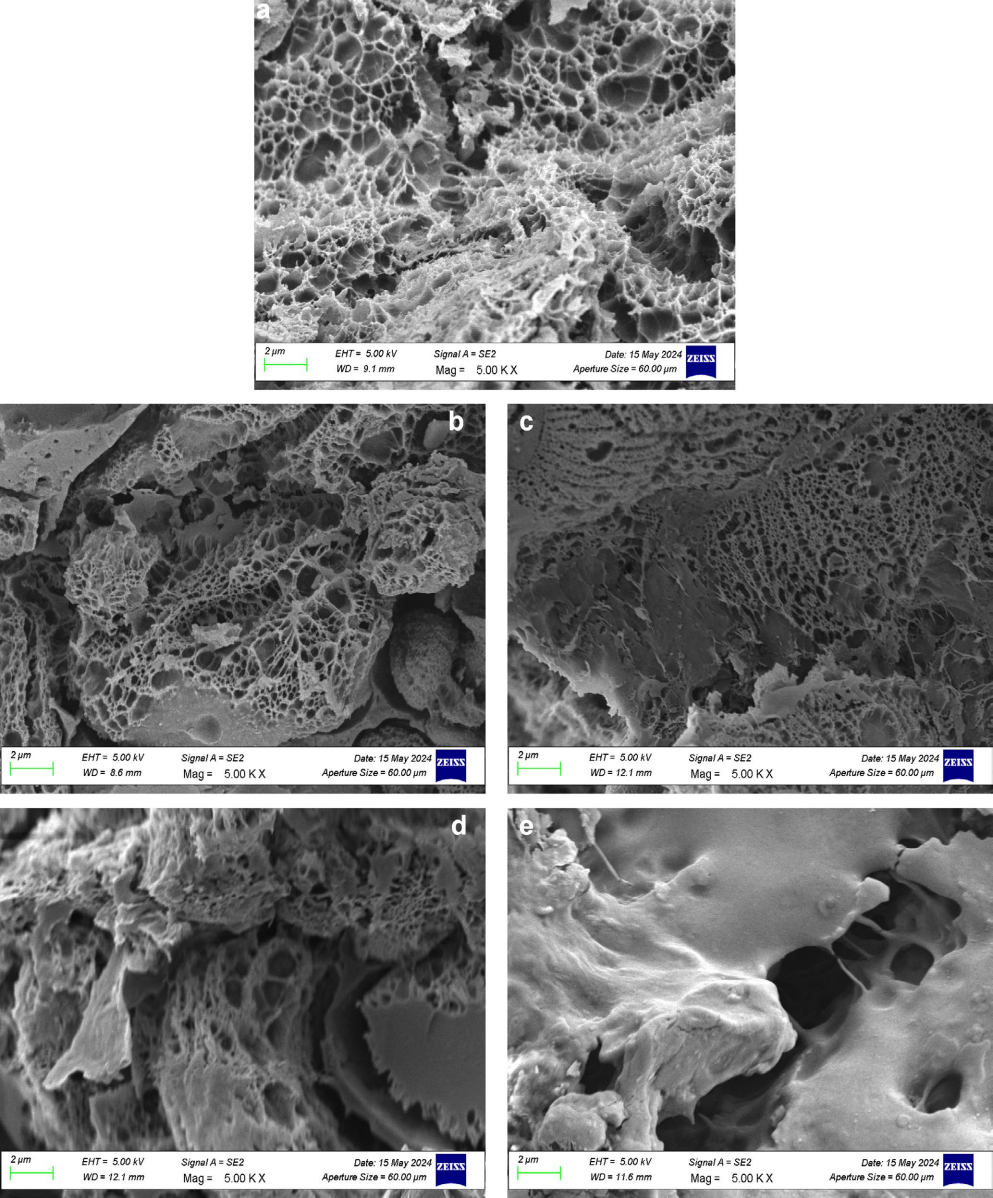
The morphological structures of breads. (a) WF‐C, (b) 12.5‐RMB, (c) 25‐RMB, (d) 12.5‐EMB, (e) 25‐EMB. 12.5‐EMB, 12.5% extruded mung bean flour‐87.5% wheat flour bread; 12.5‐RMB, 12.5% raw mung bean flour‐87.5% wheat flour bread; 25‐EMB, 25% extruded mung bean flour‐75% wheat flour bread; 25‐RMB, 25% raw mung bean flour‐75% wheat flour bread; WB‐C, wheat flour bread (control bread).

**FIGURE 8:**
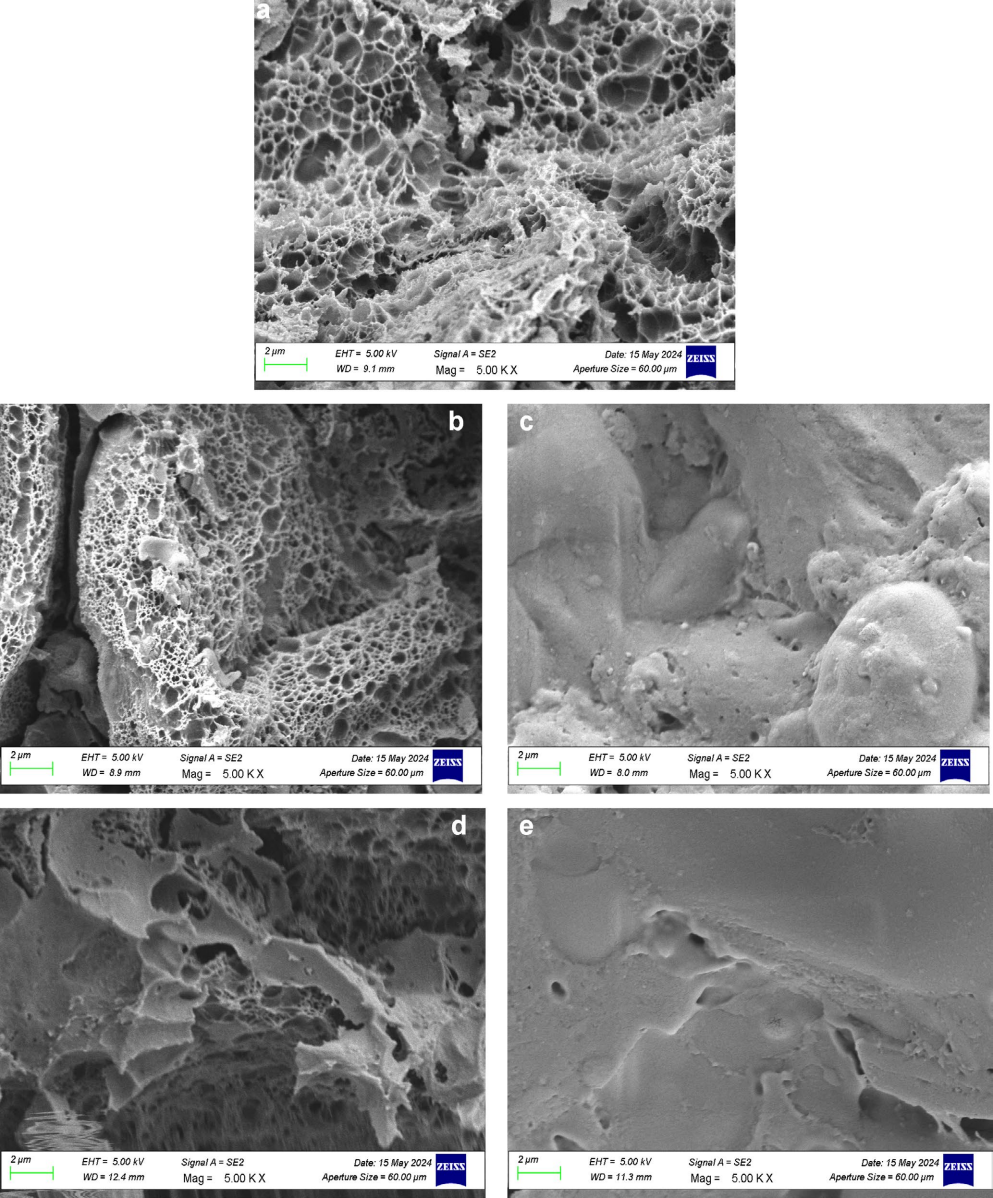
The morphological structures of breads. (a) WF‐C, (b) 12.5‐RBB, (c) 25‐RBB, (d) 12.5‐E‐BB, (e) 25‐EBB. WB‐C; Wheat flour bread (control bread). 12.5‐EBB, 12.5% extruded broad bean flour‐87.5% wheat flour bread; 12.5‐RBB, 12.5% raw broad bean flour‐87.5% wheat flour bread; 25‐EBB, 25% extruded broad bean flour‐75% wheat flour bread; 25‐RBB, 25% raw broad bean flour‐75% wheat flour bread.

The morphological properties of breads prepared with raw and extruded pulse flours were examined by scanning electron microscopy (Figures 7 and 8). The control bread sample exhibited a uniform and even porous texture typical of a well‐developed gluten network that is capable of trapping gas effectively during fermentation and baking. However, bread samples enriched with RMF and RBF had greater structural irregularities, such as larger pores with random distribution. This is perhaps because the incorporation of raw legume flours disrupts the development of gluten, resulting in lower dough elasticity and gas retention. Bread samples prepared using EMF and EBF, on the other hand, had tighter‐grained and denser textures with thicker cell walls. Partial gelatinization of starch and protein denaturation occurring during extrusion modify the water absorption and swelling capacity of the flour. As for the bread, the texture and mouthfeel are negatively affected since gas expansion will be less, and the whole product's ability to absorb and retain water will be altered. Higher pulse flour levels, regardless of processing, led to disruption of the gluten structure, as evidenced by the more inhomogeneous and fragmented pore structures in both raw and extruded systems. This was most likely a result of wheat gluten dilution with non‐gluten proteins present in broad bean and mung bean flour, impacting dough viscoelasticity and disrupting the overall structure of the gluten network. However, extrusion appears to alter the extent of this disturbance. Whereas the RMF and RBF incorporated blends resulted in a coarser, more open crumb structure, extruded flour‐based breads created a denser network. This may be explained by the altered hydration properties and the new structural interactions between gelatinized starch and denatured proteins.

## 4 Conclusion

In this study, the effect of different extrusion conditions on the techno‐functional, structural, and pasting properties of mung bean and broad bean flours and the use of extruded flours in bread production were evaluated. The optimal extrusion conditions were determined as 165°C die temperature and 200 rpm screw speed for both flours. Extrusion process increased WAI while reducing PA and ISDF content. The crystalline structure of starch was disrupted, resulting in the formation of amorphous regions. Extrusion under high shear conditions resulted in decreased PV and cold paste viscosity, indicating structural degradation of starch and altered hydration and thickening properties. Additionally, the inclusion of pulse flours affected bread quality, resulting in lower specific volume, dark crumb color, and more irregular pore structure as their ratio increased.

Based on these findings, it can be concluded that the functional properties of mung bean and broad bean flours can be significantly modified through the extrusion process, which overall enhances both the nutritional and functional qualities of these pulse flours, supporting their potential application in cereal‐based products. Pulses have also gained popularity due to their high content of macro and micronutrients, and bioactive compounds that improve the nutritional and functional properties of the final food products. Future studies will focus on using extruded pulse flours due to their great potential in different formulations such as breakfast cereals, bars, healthy drinks, and cereal‐based products.

## Author Contributions

**Kübra Tuluk:** conceptualization (equal), data curation (equal), formal analysis (equal), investigation (equal), methodology (equal), validation (equal), writing – original draft (equal), writing – review and editing (equal). **Burak Altınel:** conceptualization (equal), data curation (equal), formal analysis (equal), investigation (equal), methodology (equal), supervision (equal), validation (equal), writing – original draft (equal), writing – review and editing (equal). **Neslihan Bozdogan:** conceptualization (equal), data curation (equal), formal analysis (equal), investigation (equal), methodology (equal), validation (equal), writing – original draft (equal), writing – review and editing (equal). **Sebnem Tavman:** conceptualization (equal), supervision (equal), writing – review and editing (equal). **Seher Kumcuoglu:** conceptualization (equal), funding acquisition (equal), investigation (equal), methodology (equal), project administration (equal), resources (equal), supervision (equal), writing – original draft (equal), writing – review and editing (equal).

## Conflicts of Interest

The authors declare no conflicts of interest.

## Supporting information


**Data S1:** fsn370801‐sup‐0001‐Supinfo.docx.

## Data Availability

Data available on request from the authors.
