# Supplementary material for: The Effects of Die Temperature and Screw Speed on Extruded Pulse Flours and Their Application in Bread Production
Source: Food Sci Nutr. 2025 Aug 25;13(9):e70801. doi: 10.1002/fsn3.70801 (PMC12376178; doi:10.1002/fsn3.70801)
Supplement: Supplementary file 1 — Data S1: fsn370801‐sup‐0001‐Supinfo.docx. [file FSN3-13-e70801-s001.docx]

**Supplementary Material**

**Table S1.** ANOVA for the response variables of extruded flours.

|  | **WAI** | | **PA** | | **ISDF** | |
| --- | --- | --- | --- | --- | --- | --- |
| **Source** | **Sum of squares** | ***p*-value** | **Sum of squares** | ***p*-value** | **Sum of squares** | ***p*-value** |
| **Mung bean** |  |  |  |  |  |  |
| Model | 0.3401 | <0.0001 | 5.465E+05 | <0.0001 | 26.00 | <0.0001 |
| Linear | | | | | | |
| A | 0.1162 | <0.0001 | 4.916E+05 | <0.0001 | 21.45 | <0.0001 |
| B | 0.2238 | <0.0001 | 24565.87 | <0.0001 | 4.20 | <0.0001 |
| Interaction | | | | | | |
| AB | - | - | 88.48 | 0.1271 | 0.1098 | 0.0417 |
| Quadratic | | | | | | |
| A^2^ | - | - | 23468.05 | <0.0001 | 0.2216 | 0.0096 |
| B^2^ | - | - | 309.77 | 0.0143 | 0.0017 | 0.7675 |
| Residual | 0.0049 | - | 206.79 | - | 0.1242 |  |
| Lack of fit | 0.0026 | 0.6605ns | 155.83 | 0.1040ns | 0.0981 | 0.0771ns |
| Pure error | 0.0024 |  | 50.96 |  | 0.0262 |  |
| Total | 0.3450 |  | 5.467E+05 |  | 26.12 |  |
| R^2^ | 0.9857 |  | 0.9996 |  | 0.9952 |  |
| Adj-R^2^ | 0.9828 |  | 0.9994 |  | 0.9918 |  |
| CV | 0.6552 |  | 0.5602 |  | 1.01 |  |
|  | | | | | | |
| **Broad bean** |  |  |  |  |  |  |
| Model | 0.6609 | <0.0001 | 6.223E+05 | <0.0001 | 13.41 | <0.0001 |
| Linear | | | | | | |
| A | 0.1111 | <0.0001 | 5.836E+05 | <0.0001 | 11.98 | <0.0001 |
| B | 0.5498 | <0.0001 | 34287.48 | <0.0001 | 1.13 | 0.0008 |
| Interaction | | | | | | |
| AB | - | - | 936.44 | 0.0085 | 0.0297 | 0.3894 |
| Quadratic | | | | | | |
| A^2^ | - | - | 3424.85 | 0.0002 | 0.1447 | 0.0823 |
| B^2^ | - | - | 694.01 | 0.0170 | 0.0329 | 0.3663 |
| Residual | 0.0060 | - | 501.14 | - | 0.2466 |  |
| Lack of fit | 0.0030 | 0.6743ns | 358.96 | 0.1358ns | 0.1998 | 0.0630 |
| Pure error | 0.0029 |  | 142.17 |  | 0.0468 |  |
| Total | 0.6669 |  | 6.228E+05 |  | 13.65 |  |
| R^2^ | 0.9910 |  | 0.9992 |  | 0.9819 |  |
| Adj-R^2^ | 0.9892 |  | 0.9986 |  | 0.9690 |  |
| CV | 0.6757 |  | 0.4704 |  | 1.09 |  |

A; Die temperature. B; Screw speed. ns; Not significant..WAI; Water absorption index. PA; Phytic acid. ISDF; Insoluble dietary fibre.

**Table S2.** Proximate properties of flours.

|  | WF | RMF | EMF | RBF | EBF |
| --- | --- | --- | --- | --- | --- |
| Moisture content (%) | 12.68 ± 0.13 | 10.22 ± 0.17 | 8.27 ± 0.14 | 12.35 ± 0.22 | 8.65 ± 0.07 |
| Protein content (%)^§^ | 12.41 ± 0.03 | 25.72 ± 0.23 | 25.58 ± 0.04 | 32.72 ± 1.89 | 31.12 ± 0.12 |
| Ash content (%)^§^ | 0.56 ± 0.01 | 3.02 ± 0.03 | 3.13 ± 0.03 | 3.12 ± 0.01 | 3.35 ± 0.04 |
| Total dietary fiber content (%)^§^ | 3.72+0.08 | 18.35 ± 0.35 | 14.74 ± 0.13 | 24.24 ± 1.02 | 19.12 ± 0.10 |
| *L** | 94.82 ± 0.65 | 84.13 ± 1.15 | 66.75 ± 0.26 | 90.43 ± 0.80 | 75.51 ± 0.57 |
| *a** | 0.44 ± 0.02 | -0.45 ± 0.02 | 7.89 ± 0.14 | 0.41 ± 0.02 | 6.78 ± 0.15 |
| *b** | 11.01 ± 0.14 | 17.75 ± 0.42 | 29.14 ± 0.72 | 20.10 ± 0.43 | 34.29 ± 0.47 |

WF; Wheat flour. RMF; Raw mung bean flour. EMF; Ekstruded mung bean flour. RBF; Raw broad bean flour. EBF; Ekstruded broad bean flour. WAI; Water absorption index.

^§^: in dry matter.

Data are expressed as mean values ± standard deviations. n=4 for moisture content and L*,a*,b*. n=3 for protein content and ash content. n=2 for total dietary fiber content.
